# Booster COVID-19 mRNA vaccination ameliorates impaired B-cell but not T-cell responses in older adults

**DOI:** 10.3389/fimmu.2024.1455334

**Published:** 2024-12-09

**Authors:** Kohei Kometani, Takaaki Yorimitsu, Norihide Jo, Erina Yamaguchi, Osamu Kikuchi, Masaru Fukahori, Takeshi Sawada, Yoshitaka Tsujimoto, Ayana Sunami, Mengqian Li, Takeshi Ito, Yann Pretemer, Yuxian Gao, Yu Hidaka, Masaki Yamamoto, Natsuko Kaku, Yu Nakagama, Yasutoshi Kido, Alba Grifoni, Alessandro Sette, Miki Nagao, Satoshi Morita, Takako E. Nakajima, Manabu Muto, Yoko Hamazaki

**Affiliations:** ^1^ Department of Life Science Frontiers, Center for iPS Cell Research and Application (CiRA), Kyoto University, Kyoto, Japan; ^2^ Department of Human Health Sciences, Graduate School of Medicine, Kyoto University, Kyoto, Japan; ^3^ Alliance Laboratory for Advanced Medical Research, Graduate School of Medicine, Kyoto University, Kyoto, Japan; ^4^ Department of Medical Oncology, Graduate School of Medicine, Kyoto University, Kyoto, Japan; ^5^ Clinical Bio-Resource Center, Kyoto University Hospital, Kyoto, Japan; ^6^ Center for Cancer Immunotherapy and Immunobiology, Kyoto University, Kyoto, Japan; ^7^ Department of Early Clinical Development, Graduate School of Medicine, Kyoto University, Kyoto, Japan; ^8^ Kyoto Innovation Center for Next Generation Clinical Trials and iPS Cell Therapy (Ki-CONNECT), Kyoto University Hospital, Kyoto, Japan; ^9^ Department of Neurosurgery, Graduate School of Medicine, Kyoto University, Kyoto, Japan; ^10^ Laboratory of Immunobiology, Graduate School of Medicine, Kyoto University, Kyoto, Japan; ^11^ Department of Biomedical Statistics and Bioinformatics, Graduate School of Medicine, Kyoto University, Kyoto, Japan; ^12^ Department of Clinical Laboratory Medicine, Graduate School of Medicine, Kyoto University, Kyoto, Japan; ^13^ Department of Virology and Parasitology, Graduate School of Medicine, Osaka Metropolitan University, Osaka, Japan; ^14^ Osaka International Research Center for Infectious Diseases, Osaka Metropolitan University, Osaka, Japan; ^15^ Center for Vaccine Innovation, La Jolla Institute for Immunology (LJI), La Jolla, CA, United States; ^16^ Department of Medicine, Division of Infectious Diseases and Global Public Health, University of California, San Diego (UCSD), La Jolla, United States; ^17^ Kyoto University Immunomonitoring Center, Kyoto, Japan

**Keywords:** immunological memory, mRNA vaccine, immune aging, memory T cells, memory B cells, cTfh, booster vaccination, COVID-19 article type: original research article

## Abstract

Age-associated differences in the effect of repetitive vaccination, particularly on memory T-cell and B-cell responses, remain unclear. While older adults (aged ≥65 years) exhibited enhanced IgG responses following COVID-19 mRNA booster vaccination, they produced fewer spike-specific circulating follicular helper T cells-1 than younger adults. Similarly, the cytotoxic CD8^+^ T-cell response remained diminished with reduced PD-1 expression even after booster vaccination compared with that in younger adults, suggesting impaired memory T-cell activation in older adults. In contrast, although B-cell responses in older adults were weaker than those in younger adults in the primary response, the responses were significantly enhanced upon booster vaccination, reaching levels comparable with that observed in younger adults. Therefore, while booster vaccination ameliorates impaired humoral immunity in older adults by efficiently stimulating memory B-cell responses, it may less effectively enhance T-cell-mediated cellular immunity. Our study provides insights for the development of effective therapeutic and vaccine strategies for the most vulnerable older population.

## 1 Introduction

Advanced age is the most significant risk factor for various infectious diseases, including coronavirus disease (COVID-19) (1–3), mainly because of the age-associated decline in immune competence caused by various factors, including thymic involution, chronic diseases, and frailty. Therefore, vaccination is strongly recommended for older adults; however, the benefits and efficacy of vaccination are limited, primarily because of the decreased effectiveness of adaptive immunity (4–6). While the newly developed mRNA vaccines for severe acute respiratory syndrome coronavirus 2 (SARS-CoV-2) are highly effective at preventing severe illness and infection even in older adults (7), peak IgG levels and neutralizing antibody titers are significantly lower after two doses of vaccination, resulting in older adults becoming vulnerable earlier post-vaccination (8–11). Detailed immunological studies, including our investigations, have revealed that both T-cell and B-cell responses in older adults are lower than those in younger individuals, explaining the lower vaccine efficacy in older population (8, 12).

Importantly, after the third dose, antibody titers and neutralizing activity in older adults become comparable with those in younger adults (13–15), likely because of the effect of immunological memory. After the primary responses, the adaptive immune system generates memory T cells and memory B cells (MBCs), which facilitate a fast and strong response upon re-exposure to the same antigens (16–18). Previous studies have suggested that the memory responses of T cells, which cross-recognize variant strains (19–22), may occur even in older adults after the third or higher dose (23, 24). However, the characteristics of the CD8^+^ T-cell response, which decline more strongly with age than those of the CD4^+^ T-cell response and are highly related to asymptomatic SARS-CoV-2 infection (25) in older adults, remain unclear. Moreover, the detailed mechanisms of the booster effects on antibody responses and age-related differences in T-cell and B-cell memory responses have not been elucidated.

Notably, the mechanisms of lifelong maintenance of T-cell and B-cell compartments are substantially different. Although T cells play central roles in antigen-specific antibody responses and cytotoxicity against virus-infected cells (26), the production of new T cells begins to decline during the early period of life because of thymic involution. However, the T-cell compartment is maintained long-term through continuous stimulation by self-peptide/MHC complex ligands and/or homeostatic cytokines, leading to their qualitative and compositional changes and functional dysregulations with age (27–33). In contrast, B cells are stably generated from the bone marrow throughout life (34), and MBCs or long-lived plasma cells are maintained based on competition with newly generated memory B/plasma cells for survival factors and niches (35–37). Although Tfh cells facilitate the differentiation of B cells into plasma cells and have a pivotal role in the affinity maturation of germinal center B cells (38), studies using mouse models and *in-vitro* studies using human primary B cells have suggested that memory B-cell responses do not require CD4^+^ T cells (39–41). However, it remains unclear whether this holds true for humans *in vivo*. Importantly, mRNA vaccines are becoming a crucial vaccine modality for infectious diseases and even cancers, and their routine administration is expected, as in the case of influenza. Therefore, understanding the characteristics and detailed trajectories of memory T-cell and B-cell responses that occur after the booster vaccination in older adults, who are the most vulnerable to infectious diseases, is fundamental.

In this study, we investigated these key questions by comparing the long-term kinetics of primary (following the first and second doses) and secondary/memory (following the third dose) spike-specific CD4^+^ and CD8^+^ T-cell and B-cell responses to COVID-19 mRNA vaccination between adults (<65 years) and older adults (≥65 years) in a Japanese cohort of healthy individuals. Our findings will improve the understanding of age-related changes in memory responses in both the T-cell and B-cell subsets and are relevant for future vaccine strategies, particularly for the highly vulnerable older population.

## 2 Materials and methods

### 2.1 Study design

**Figure 1:**
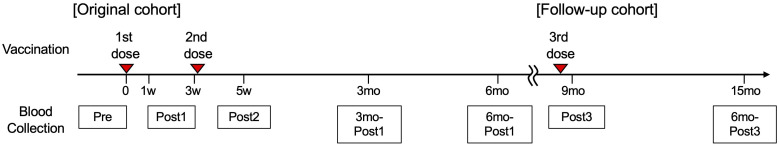
Study design. Timeline of the vaccinations and blood collections. Participants received the first dose of BNT162b2 on day 0, the second dose around day 21, and the third dose approximately nine months after the first dose. The sampling points were set before the first vaccination (Pre), seven days after the first dose to one day before the second dose (Post1), two weeks after the second dose ± four days (Post2), three months after the first dose ± two weeks (3mo-Post1), and six months after the first dose ± two weeks (6mo-Post1). The sampling points in the follow-up study were set at two weeks ± four days after the third dose (Post3) and six months ± four weeks after the third dose vaccination (6mo-Post3).

This study was reviewed and approved by the Ethics Committee of Kyoto University Graduate School and Faculty of Medicine (R0418). At the time of enrollment, all donors provided written informed consent in accordance with the Declaration of Helsinki. Donor recruitment and eligibility criteria were as previously reported (12). All donors were required to be ≥20 years of age and without any ongoing severe medical conditions, including cancer or gastrointestinal, liver, kidney, cardiovascular, hematological, or endocrine diseases. Individuals taking medications that may affect the immune system, including steroids and immunomodulatory drugs, were excluded. Only individuals scheduled to receive Pfizer BNT162b2, which comprises mRNA of the spike protein of the original Wuhan strain, were eligible at enrollment. A total of 225 individuals were enrolled in the original cohort. Six individuals did not meet the eligibility criteria, and 107 adults (aged <65 years, workers at Kyoto University Hospital) and 109 older adults (aged ≥65 years, general population) were analyzed. Blood samples were collected before vaccination (Pre), after the first dose (Post1), after the second dose (Post2), 3 months after the first dose (3mo-Post1), and 6 months after the first dose (6mo-Post1) (**Figure 1**) at the Kyoto Innovation Center for Next Generation Clinical Trials and iPS Cell Therapy and Clinical Bio-Resource Center at Kyoto University Hospital. Samples were de-identified with an anonymous code. Two participants were lost to follow-up (**Supplementary Figure 1**). To assess memory responses, we set up a follow-up study. Donors were recruited from the original cohort and provided written informed consent. In total, 208 individuals, including 103 adults and 105 older adults, were enrolled (**Supplementary Figure 1**). Participants received the third dose approximately 9 months after the first dose (**Figure 1**). During the follow-up study, samples were collected after the third dose (Post3) and six months after the third dose (6mo-Post3) (**Figure 1**). Samples from donors who met the exclusion criteria (e.g., lack of a third vaccination, vaccination with mRNA-1273 or an unspecified third vaccine, delayed third vaccination, receipt of a fourth vaccination, or a history of infection) were excluded from analysis at the time of the respective event or thereafter.

### 2.2 Peripheral blood mononuclear cell isolation, cryopreservation, and thawing

Whole blood was collected into Vacutainer CPT cell preparation tubes with sodium citrate (BD Biosciences) according to the manufacturer’s instructions. The samples were centrifuged at 1500 × *g* at room temperature for 20 min, and peripheral blood mononuclear cells (PBMCs) were collected. The isolated PBMCs were resuspended in CELLBANKER 1 (Zenogen Pharma) at 8 × 10^6^ cells ml^–1^, stored at –80°C for at least 24 h, and transferred into a liquid nitrogen storage tank until use. Cryopreserved PBMCs were thawed in pre-warmed X-VIVO15 (Lonza) without serum. After centrifugation, the cells were washed with X-VIVO15 once and used directly in assays.

### 2.3 Complete blood counts

Whole blood was collected in ethylenediaminetetraacetic acid-2Na tubes and analyzed using an Automated Hematology Analyzer XN-9000 (Sysmex) at the Department of Clinical Laboratory, Kyoto University Hospital.

### 2.4 Serology

Whole blood was collected in Venoject VP-P075K (Terumo) blood collection tubes for serum isolation. The tubes were centrifuged at 1100 × *g* at 4°C for 4 min. Serum anti-SARS-CoV-2 nucleocapsid IgM/IgG antibody levels were measured using an Elecsys Anti-SARS-CoV-2 assay on a cobas 8000 system (Roche Diagnostics KK) at the Department of Clinical Laboratory, Kyoto University Hospital. Anti-SARS-CoV-2 receptor-binding domain (RBD) IgM and IgG antibodies were measured at LSI Medience (Tokyo, Japan) using ARCHITECT SARS-CoV-2 IgM and ARCHITECT SARS-CoV-2 IgG II Quant (Abbott), respectively. The cutoff values for anti-SARS-CoV-2 (N protein) IgM/IgG, anti-SARS-CoV-2 RBD IgM, and IgG were 0.8 cutoff index (COI), 1.0 (COI), and 50 (AU ml^–1^), respectively.

### 2.5 Surrogate virus neutralization assay

Neutralizing antibodies in the serum against the RBD of the spike protein of the Wuhan, Delta, and Omicron strains were measured using a SARS-CoV-2 surrogate virus neutralization test and horseradish peroxidase-conjugated strain-specific recombinant RBD protein (both from GenScript) according to the manufacturer’s instruction. The percentage inhibition was calculated using the following formula:


$$\begin{array}{c} \%\text{ inhibition}=(1-[\text{absorbance at }450\text{ nm }(\text{A}450)\text{ of sample}]/\\ {}[\text{A}450\text{ of negative control sample}])\times 100 \end{array}$$

### 2.6 Activation-induced marker assay

Thirty-two adults and 35 older adults for whom samples were available at all time points (Post1, Post2, 3mo-Post1, 6mo-Post1, Post3, and 6mo-Post3) were selected for flow-cytometric analysis (**Supplementary Figure 2A**). Thawed PBMCs were cultured in 100 μL of X-VIVO15 medium supplemented with 5% human AB serum (Sigma-Aldrich) in the presence of SARS-CoV-2 peptide pools (0.6 nmol ml^−1^, Miltenyi Biotech), which consisted of 15-mer peptides overlapping with 11 amino acids and covering the entire protein-coding sequence (amino acids 5–1,273) of the SARS-CoV-2 spike glycoprotein (GenBank MN908947.3, Protein QHD43416.1), in 96-well U-bottom plates (Corning) at 1 × 10^6^ cells per well at 37°C for 23 h. An equal volume of distilled water (DW) was used as a negative control. After stimulation, the cells were stained with fluorochrome-conjugated surface antibodies in the presence of FcR blocking reagent (Miltenyi Biotech) at 4°C for 30 min. The antibodies used in the Activation-induced marker (AIM) assay are listed in **Supplementary Table 1**. The cells were washed and stained with Ghost Dye Red 710 (Tonbo Biosciences) to distinguish viable cells from dead ones. After a final wash, the cells were resuspended in phosphate-buffered saline (PBS) with 2% fetal bovine serum (FBS) and analyzed using a Northern Lights 3000 flow cytometer (Cytek Biosciences). The data were analyzed using the FlowJo software (BD Biosciences). Spike-specific AIM^+^ T cells were defined based on the coexpression of CD134 (OX40) and CD137 (4-1BB) for CD4^+^ T cells and of CD69 and CD137 for CD8^+^ T cells (**Supplementary Figure 3**). Antigen-specific responses, including AIM^+^CD4^+^ T cells, AIM^+^cTfh1 cells, and AIM^+^CD8^+^ T cells, were quantified as the frequency (%) of AIM^+^ cells stimulated with SARS-CoV-2 peptide after subtracting that of AIM^+^ cells in the corresponding negative control cells cultured with DW. The limit of detection (LOD) of AIM^+^ cells was calculated as previously described (42). The LOD values for AIM^+^CD4^+^ T cells, AIM^+^ cTfh1 cells, and AIM^+^CD8^+^ T cells were 0.03448%, 0.0028173%, and 0.05975%, respectively. Absolute numbers of AIM^+^ cells in blood were calculated based on the cell numbers by blood count and the frequency of AIM^+^ cells. The stimulation Index (SI) was calculated by dividing the percentage of AIM^+^ cells after SARS-CoV-2 peptide pool stimulation by that after DW treatment. If the percentage of AIM^+^ cells upon DW stimulation was 0, the minimum value was used instead of 0. Samples with SI > 2 were used for phenotypic analysis of antigen-specific T cells including geometric mean fluorescence intensity of PD-1, and characterization of T-cell subsets.

To evaluate the reactivity to a variant strain, 6.7 × 10^5^ PBMCs were cultured in the presence of ancestral strain or the Omicron variant megapool, composed of overlapping 15-mers peptides (with 10 amino acid overlaps), resuspended in DMSO as previously reported (1 μg ml^–1^) (22) in 96-well U-bottom plates. An equal amount of DMSO was used in negative control samples. Five adults and five older adults were randomly selected for flow-cytometric analysis (**Supplementary Figure 4C**).

### 2.7 Flow-cytometric detection of spike-specific B cells

SARS-CoV-2 spike-specific B cells were detected using biotinylated recombinant spike protein and fluorochrome-conjugated streptavidin (SAv). Biotinylated recombinant ancestral spike protein (R&D Systems) was mixed with SAv-BV421 or SAv-BV711 (BioLegend). Biotinylated recombinant Omicron spike protein (R&D Systems) was mixed with SAv-BV605 or SAv-BV785 (BioLegend). d-Biotin (Nacalai Tesque) was mixed with SAv-BV510. d-Biotin/SAv-BV510 probe was used as a decoy probe to gate out non-specific cells (**Supplementary Figure 6**). All recombinant probe proteins and SAv-fluorochrome were mixed at a 25:1 mass ratio and incubated at 4°C for 2 h. For staining, thawed PBMCs were incubated with FcR blocking reagent (Miltenyi Biotech) at 4°C for 10 min. The cells were washed with PBS containing 2% FBS, followed by incubation with a probe master mix at 4°C for 1 h. The cells were washed and stained with anti-CD19, anti-CD20, anti-CD21, anti-CD27, anti-CD38, anti-IgM, anti-IgD, and anti-IgG (BioLegend; **Supplementary Table 1**) at 4°C for 20 min. The cells were washed again with PBS and incubated with Ghost Dye Red 710 (Tonbo Biosciences) at 4°C for 30 min. After a final wash with PBS containing 2% FBS, the cells were resuspended in the PBS solution and analyzed using the Northern Lights 3000 flow cytometer (Cytek Biosciences) and FlowJo software (BD Bioscences).

To evaluate the B-cell receptor (BCR) affinity of spike-binding MBCs, the MFIs of ancestral spike-SAv/BV421 or Omicron spike-SAv/BV785 within spike-binding MBCs were used as an indicator (43–46).

### 2.8 Statistical analysis

Statistical analyses were performed using GraphPad Prism 9. Details on statistics, including group sizes, the statistical tests used, and significance values, are provided in the figure legends. All statistical tests (Mann–Whitney test, Wilcoxon matched-pairs signed rank test, and Spearman’s rank correlation) were two-sided, and *p-*values < 0.05 were considered statistically significant. According to Guilford’s rule of thumb, a correlation coefficient (r_s_) of ≥ 0.2 and *p* < 0.05 were considered to indicate a correlation (47). Nonparametric statistical tests were used because the data were not assumed to be normally distributed. No statistical methods were used to predetermine the sample size, but the sample size of original cohort in this study was similar to that in previous reports (8, 48). As this was an observational study, randomization was not applied. The investigators were not blinded to allocation during the study or outcome assessment.

## 3 Results

### 3.1 IgG response following booster vaccination is comparable between adults and older adults

We previously investigated T-cell and antibody responses during the induction and contraction phases of primary immune responses following two doses of BNT162b2 vaccination in adults (age <65 years, n = 107) and older adults (age ≥65 years, n = 109) (Pre; median, -14 days [range, -29 to 0 days], Post1; median, 11 days [range, 6-21 days], Post2; median, 34 days [range, 30-39 days], and 3mo-Post1; median, 93 days [range, 77-104 days]) [**Figure 1**, **Table 1A**, and **Supplementary Figure 1**)] (12). To explore age-related differences in the maintenance of memory T and B cells and their memory responses, PBMCs were additionally collected six months after the first dose (6mo-Post1; median, 180 days [range, 170–195 days]). In a follow-up study, we recruited 208 individuals from the original cohort (**Figure 1**, **Table 1B**, and **Supplementary Figure 1**), comprising adults (n = 103) and older adults (n = 105). Blood samples were collected two weeks following the third dose (Post3; median, 269 days [range, 239–295 days]) and six months after the third dose [6mo-Post3; median, 416 days (range, 317–444 days)].

**Table 1:** Participant characteristics for antibody response analysis.

(A) Original cohort				
			Adults (<65 years) n=107	Older Adults (≥65 years) n=109
Age (years)	Median (range)		43 (24–63)	71 (65–82)
Sex	n (%)	Male	43 (40.2%)	56 (51.4%)
		Female	64 (59.8%)	53 (48.6%)
(B) Follow-up cohort				
			Adults (<65 years) n=103	Older Adults (≥65 years) n=105
Age (years)	Median (range)		42 (24–63)	71 (65–82)
Sex	n (%)	Male	43 (41.7%)	55 (52.4%)
		Female	60 (58.3%)	50 (47.6%)

**Figure 2:**
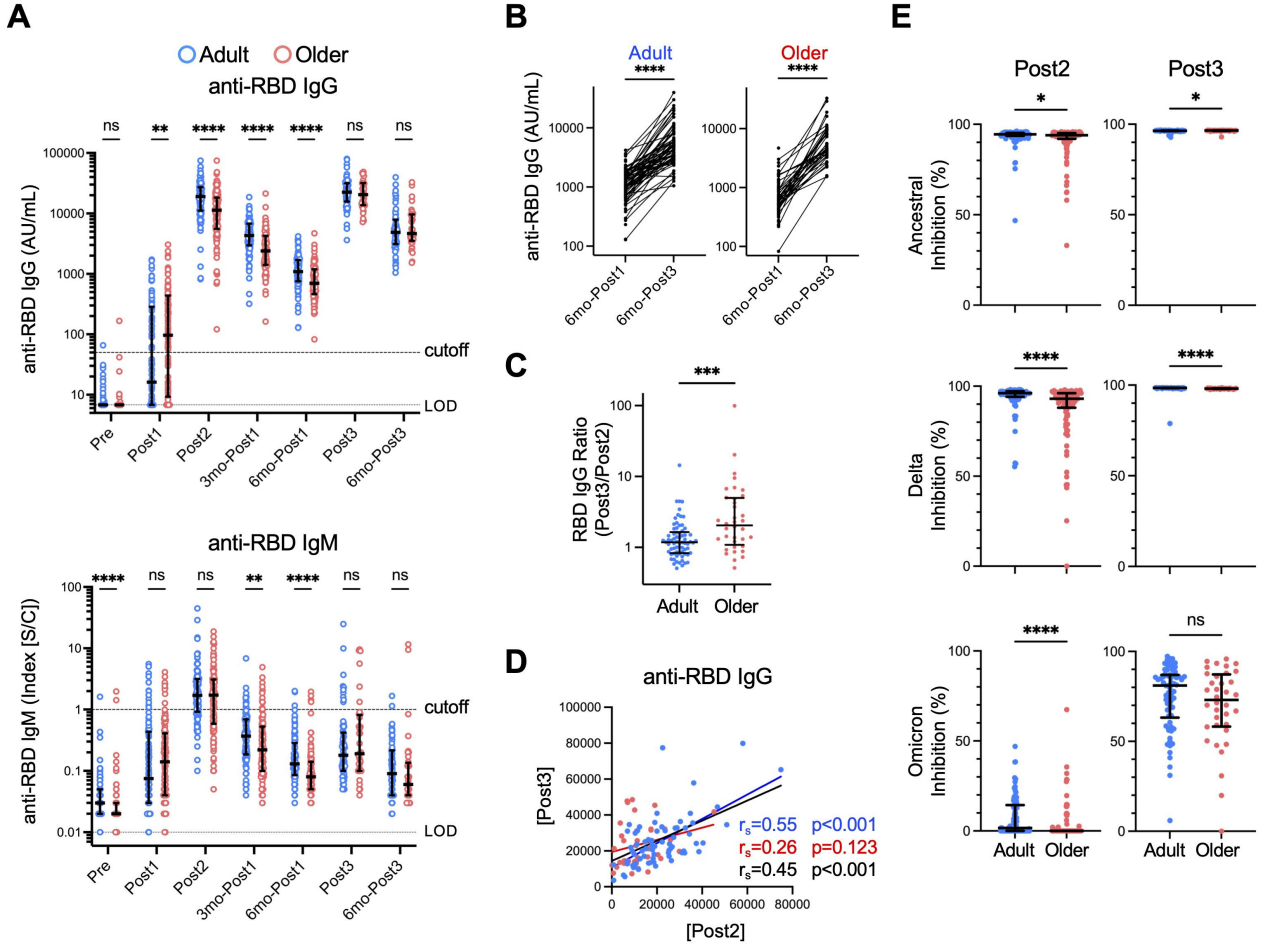
Anti-RBD antibody responses to third-dose vaccination are comparable between adults and older adults. **(A)** Concentrations of anti-RBD IgG and IgM antibodies. Each dot represents the serum antibody titer of each subject. Center lines and error bars indicate the median and interquartile range (IQR), respectively. Dashed and dotted lines indicate cutoffs and limits of detection (LODs), respectively. Sample numbers were as follows: 107 (Adults) and 108 (Older adults) (Pre), 106 and 108 (Post1), 105 and 108 (Post2), 105 and 106 (3mo-Post1), 105 and 106 (6mo-Post1), 70 and 36 (Post3), and 65 and 37 (6mo-Post3), respectively. (See also **Supplementary Figure 1**). **(B)** Anti-RBD IgG antibody concentrations six months after the first dose (6mo-Post1) and six months after the third dose (6mo-Post3). ****p < 0.0001 (Wilcoxon matched-pairs signed rank test). **(C)** The ratio of the anti-RBD IgG titer after the third vaccination (Post3) to that after the second dose (Post2). **(D)** Correlation between anti-RBD IgG titers (AU/mL) after the second dose (Post2) and after the third dose (Post3). Spearman’s rank correlation (r_s_) was used to identify correlations between two variables, and regression lines were drawn based on linear regression analysis. Blue, red, and black characters represent the results of adults, older adults, and both groups, respectively. **(E)** Neutralization capacity of sera collected after the second dose (Post2) and the third dose (Post3) against RBD of ancestral, Delta, and Omicron strains. Center lines and error bars indicate the median and IQR, respectively. **(A, C, E)** Blue and red represent adults and older adults, respectively. *p < 0.05, **p < 0.01, ***p < 0.001, ****p < 0.0001, ns, not significant (Mann–Whitney test).

We first examined antibody responses by measuring anti- RBD IgM and IgG titers. Samples from donors who experienced infection, did not receive a third dose, received an additional dose, underwent unspecified or different types of vaccination (e.g., mRNA-1273), or experienced delayed administration of the third dose were excluded at the time of the respective event or thereafter. History of COVID-19 was defined by self-report or SARS-CoV-2 anti-nucleocapsid antibody titer ≥ 0.8 (**Supplementary Figure 1**). As reported in previous studies, including ours (12, 13), anti-RBD IgG titers were lower in older adults than in adults after the second dose (Post2), and this tendency was maintained for 6 months (6mo-Post1) (**Figure 2A**). In contrast, the third-dose vaccination resulted in a notable increase in anti-RBD IgG concentrations in both groups (Post3), with the antibody titers in older adults reaching the same levels as those in adults, and this effect also persisted for up to 6 months after the third dose (**Figure 2A**) (13–15). Anti-RBD IgM levels showed a minimal increase following the third dose in both groups, which was expected in the context of memory response (49). As for antibody maintenance, the levels of anti-RBD IgG antibody at 6 months following the third booster vaccination (6mo-Post3) were significantly higher than those following the initial vaccination (6mo-Post1) in both groups (**Figure 2B**) (median [interquartile range (IQR)]: adults: 1090 [938.0], older adults: 699.5 [735.5] [6mo-Post1]; adults: 4860 [4750], older adults: 4660 [6115] [6mo-Post3]). The increase in antibody titers from the second to the third dose was significantly higher in older adults than in adults (**Figure 2C**). We also examined the correlations between primary and memory responses. To explore individual variation independent of age, we analyzed the results for both age-specific groups as well as the combined group. Given the observed correlation between IgG titers after the second (Post2) and third (Post3) doses (**Figure 2D**), individuals who exhibited a favorable response to the initial vaccination were likely to also have a better response to the booster vaccination. The lack of correlation in the older adult group (**Figure 2D**) was possibly because even those with low titers after the initial vaccination often had raised IgG titers following the booster dose. Together, these results indicate that the third-dose vaccination effectively stimulates IgG production in older adults to levels comparable with those in adults.

Next, we sought to evaluate anti-RBD IgG quality, which is regulated by the germinal center reaction (50). To this end, we measured the activity of neutralizing antibodies against the RBDs of both ancestral and mutated strains using a surrogate virus neutralization assay. Sera from 95.2% and 82.4% of adult and older adult donors exhibited >90% inhibition of the Wuhan strain and 89.5% and 69.4% inhibition of Delta variants, respectively, after the second dose, whereas 0.0% and 0.0% of donor sera inhibited the Omicron variant, which has more mutations (**Figure 2E**). Following the third dose, sera from most (>98%) donors in both groups inhibited the Wuhan and Delta strains by >90% (**Figure 2E**). Moreover, neutralizing activity against the Omicron variant was significantly increased, as previously reported (51, 52), and notably, there was no significant difference between the two groups (**Figure 2E**).

These results strongly suggest that booster vaccination effectively stimulates IgG production and the germinal center reaction in older adults, thus ameliorating the relatively low antibody response observed after initial vaccination.

### 3.2 Memory circulating follicular helper T cell-1 response is lower in older adults than in adults

To investigate the cellular mechanism underlying the enhanced antibody response after the third dose in older adults, we compared the memory cTfh1 response of the two groups throughout the study. PBMC samples that were available at all time points (Post1, Post2, 3mo-Post1, 6mo-Post1, Post3, and 6mo-Post3) were selected (adults, n = 32; older adults, n = 35; **Table 2**. Samples of individuals who met the exclusion criteria, including infection, were excluded at the time of the event and thereafter (**Supplementary Figure 2A**). The samples from the selected donors exhibited similar anti-RBD IgG and IgM responses as those from the original cohort (**Supplementary Figure 2B**). Vaccine-induced spike-specific T cells were quantified and characterized using AIM assays (53). PBMCs were stimulated with overlapping peptide pools derived from the complete sequence of the SARS-CoV-2 spike protein, which was used as the antigen for BNT162b2. The markers used for flow-cytometric analyses and gating strategies are shown in **Supplementary Table 1** and **Supplementary Figure 3**, respectively.

**Table 2:** Participant characteristics for memory T/B cell analysis.

			Adults (<65 years) n=32	Older Adults (≥65 years) n=35
Age (years)	Median (range)		45 (26–62)	72 (65–82)
Sex	n (%)	Male	10 (31.3%)	17 (48.6%)
		Female	22 (68.8%)	18 (51.5%)

**Figure 3:**
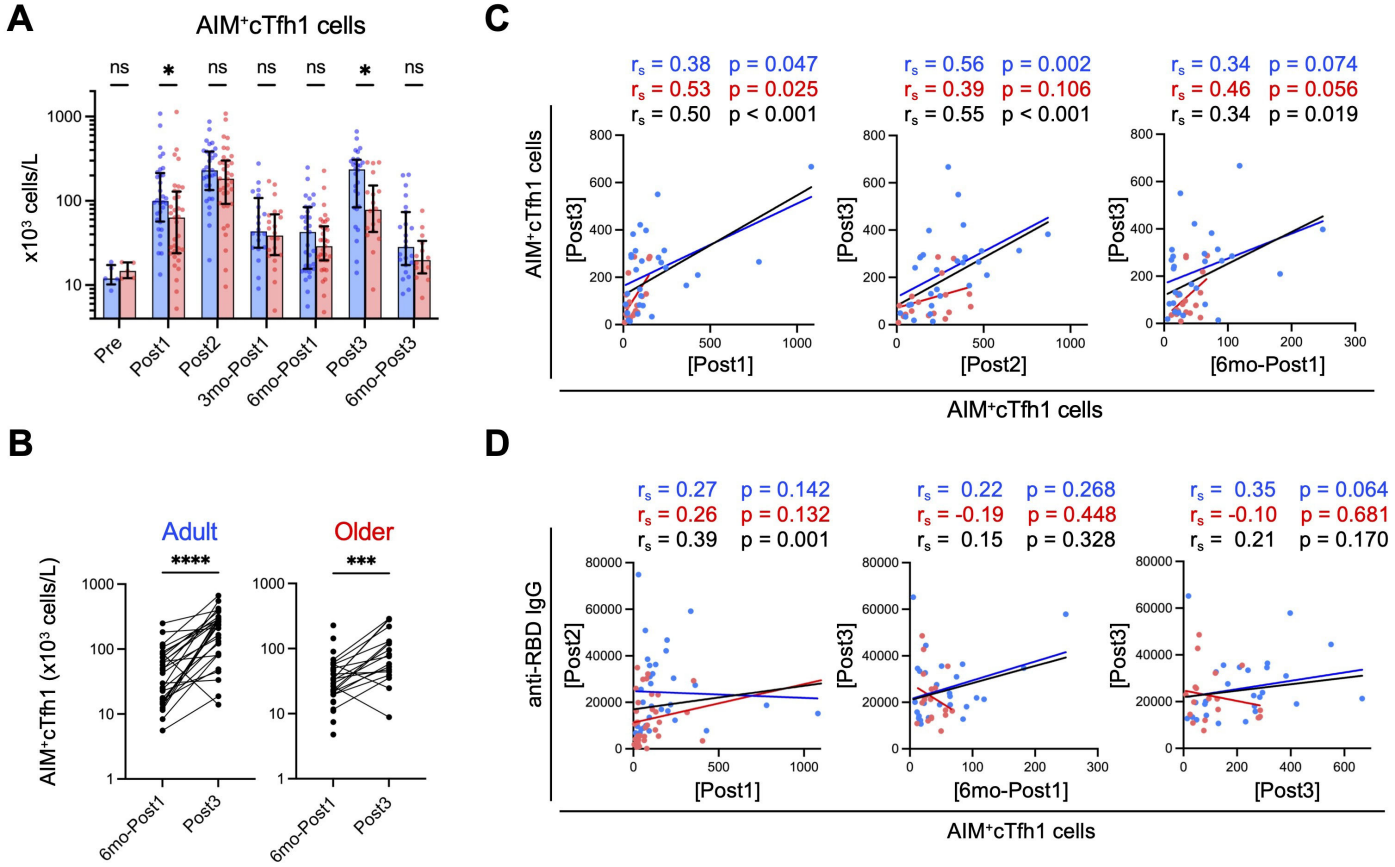
Spike-specific cTfh1 cells are fewer in older adults than in adults after third-dose vaccination. **(A)** Absolute numbers of AIM^+^cTfh1 cells in blood. Each dot represents the value of each subject. Bar graphs indicate medians, error bars indicate IQRs. Blue and red represent adults and older adults, respectively. Sample numbers were as follows: 5 (Adults) and 5 (Older adults) (Pre), 32 and 35 (Post1), 31 and 35 (Post2), 20 and 22 (3mo-Post1), 31 and 35 (6mo-Post1), 28 and 18 (Post3), and 23 and 14 (6mo-Post3), respectively. (See also **Supplementary Figure 2**). *p < 0.05, ns, not significant (Mann–Whitney test). **(B)** Comparison of the numbers (x10^3^ cells/L) of AIM^+^cTfh1 cells at six months (6mo-Post1) and after the third dose (Post3). ***p < 0.001, ****p < 0.0001 (Wilcoxon matched-pairs signed rank test). **(C)** Correlations between AIM^+^cTfh1 cell numbers (x10^3^ cells/L) at the indicated time points. **(D)** Correlations between AIM^+^cTfh1 cell numbers (x10^3^ cells/L) and anti-RBD IgG concentrations (AU/mL) at the indicated time point. **(C, D)** Spearman’s rank correlation (r_s_) was used to identify correlations between two variables, and regression lines were drawn based on linear regression analysis. Blue, red, and black characters represent the results of adults, older adults, and both groups, respectively.

The first-dose vaccination induced lower levels of spike-specific AIM^+^ cTfh1 cells (CD3^+^CD4^+^OX40^+^CD137^+^CD45RA^–^CXCR5^+^CXCR3^+^CCR6^–^) in older adults than in adults; however, the numbers peaked to similar levels in both groups following the second dose and decreased at three months (**Figure 3A**), which is consistent with previous findings (12). Even at 6 months (6mo-Post1), spike-specific CD4^+^ T cells, as well as cTfh1 cells, were well-maintained and at higher levels than those observed pre-vaccination, with no significant difference between the two groups (**Figure 3A**, **Supplementary Figure 4A**). The third dose elicited a significant increase in AIM^+^ cTfh1 cells in both groups (**Figure 3B**). However, notably, the numbers of AIM^+^ cTfh1 cells in older adults were significantly lower than those in adults (median [IQR]: adults: 236.6 [223.8]; older adults: 78.4 [109.0] ×10^3^ cells L^–1^) (**Figure 3A**). Following the third dose (Post3), AIM^+^ cTfh1 cell numbers were correlated with those after the first (Post1) and second (Post2) doses and with those before the third dose (6mo-Post1) in the combined group (**Figure 3C**). This finding suggests that individuals who exhibited a higher cTfh1 response to the initial vaccination tended to induce higher levels of cTfh1 cells following the booster vaccination. As for the phenotypes, vaccine-induced CD4^+^ T cells mostly exhibited the CCR7^+^CD45RA^–^ central memory phenotype, even after the third dose, in both groups (**Supplementary Figures 3**, **4B**), thus indicating no exhaustion of CD4^+^ T cells by booster vaccination. We did not observe a significant age-related decrease in the reactivity against variant strains, which is generally well retained in T cells (19–22), after the second or third dose (**Supplementary Figure 4C**).

Next, we examined the relationship between spike-specific cTfh1 cells and antibody titers in the combined group. In the primary response, anti-RBD IgG concentrations (Post2) correlated with the proportions of AIM^+^ cTfh1 cells (Post1) (**Figure 3D**), as previously reported (12, 54). However, in the memory response, anti-RBD IgG titers (Post3) did not correlate with AIM^+^ cTfh1 cell numbers after the third dose (Post3) (**Figure 3D**). Pre-booster memory CD4^+^ T-cell frequency is reportedly a good indicator of memory response (55); however, we observed no correlation between IgG levels after the third dose (Post3) and the frequency of AIM^+^ cTfh1 cells before the third dose (6mo-Post1), irrespective of age (**Figure 3D**).

These results indicate that older adults exhibit a lower cTfh1 response even after the booster dose. Therefore, enhanced memory IgG responses in older adults following the booster dose may not be due to the increased help from cTfh1 cells.

### 3.3 Attenuated memory CD8^+^ T-cell response in older adults is associated with impaired activation

**Figure 4:**
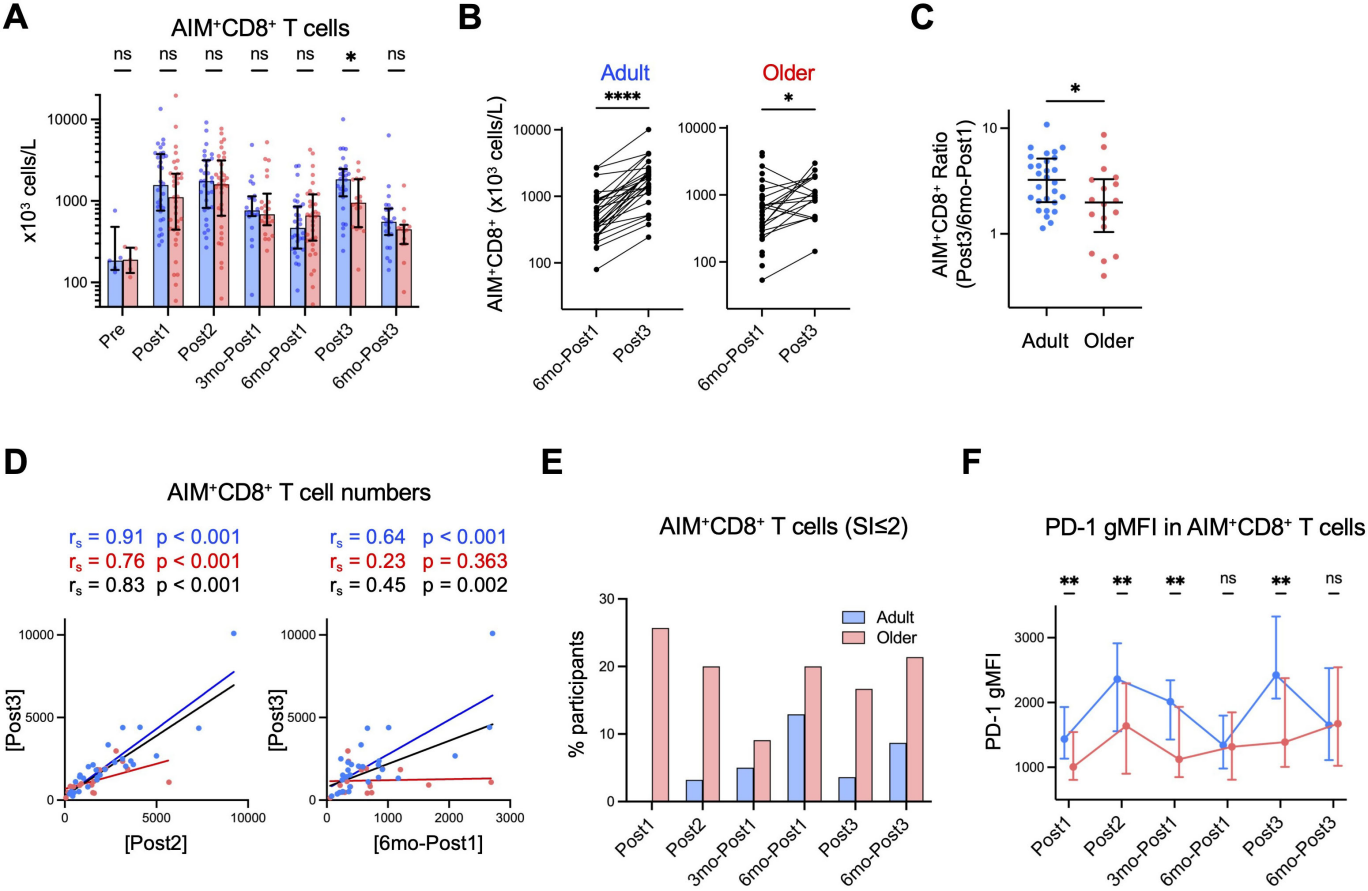
Spike-specific CD8^+^ T-cell responses are lower in older adults after the third dose. **(A)** Absolute numbers of AIM^+^CD8^+^ T cells in blood. Each dot represents the value of each subject. Bar graphs indicate medians, error bars indicate IQRs. Blue and red represent adults and older adults, respectively. **(B)** Comparison of the numbers of AIM^+^CD8^+^ T cells at six months after the first dose (6mo-Post1) and after the third dose (Post3). *p < 0.05, ****p < 0.0001 (Wilcoxon matched-pairs signed rank test). **(C)** The ratio of the AIM^+^CD8^+^ T cell number after the third vaccination (Post3) to that six months after the first dose (6mo-Post1). **(D)** Correlation between AIM^+^CD8^+^ T cell numbers at the indicated time point. Spearman’s rank correlation (r_s_) was used to identify correlations between two variables, and regression lines were drawn based on linear regression analysis. Blue, red, and black characters represent the results of adults, older adults, and both groups, respectively. **(E)** Proportion of participants with SI of AIM^+^CD8^+^ T cells ≤2 at each time point. **(F)** Geometric mean fluorescence intensity (gMFI) of PD-1 on AIM^+^CD8^+^ T cells at each time point. Blue and red represent adults and older adults, respectively. Dots and error bars indicate medians and IQRs, respectively. **(A, C, F)** *p < 0.05, **p < 0.01, ns, not significant (Mann–Whitney test).

We investigated the memory CD8^+^ T-cell response. The numbers of AIM^+^ spike-specific CD8^+^ T cells in older adults after the first dose (in the primary response) tended to be lower than those in adults, although the difference did not reach statistical significance in this study likely because of the limited sample size (n=67) compared to the previous study (n=216) (12) (**Figure 4A**). After the second dose, AIM^+^ CD8^+^ T cells reached similar levels in both groups and declined thereafter, and notably, they decreased more sharply after six months (6mo-Post1) (**Figure 4A**) than AIM^+^ CD4^+^ T cells (**Supplementary Figure 4A**). Following the third dose, AIM^+^ CD8^+^ T cells significantly increased, albeit to significantly lower levels in older adults than in adults (**Figures 4A–C**). The numbers of AIM^+^ CD8^+^ T cells following the third dose (Post3) correlated with those after the second dose (Post2) or at six months after the initial vaccination (6mo-Post1) in the combined group (**Figure 4D**). This finding suggests that individuals who exhibited a higher CD8^+^ T-cell response in the primary response tended to show a higher memory response. We observed no correlation in AIM^+^ CD8^+^ T cell numbers between 6mo-Post1 and Post3 in older adults, likely because of the inefficient induction of a CD8^+^ T-cell response following the third dose (**Figure 4D**).

As the AIM assay tends to show high background for CD8^+^ T cells (12), we additionally evaluated the CD8^+^ T-cell response using SI of AIM^+^ (%) CD8^+^ T cells. Individuals with SI value >2 are interpreted as responders (22, 56). The proportion of non-responders (SI ≤2) was higher in older adults (∼20%) than in adults (<5%) even after the third booster dose, indicating a limited CD8^+^ memory T-cell response in older adults (**Figure 4E**). Phenotypically, spike-specific CD8^+^ T cells exhibited lower proportions of central memory cells and higher proportions of terminally differentiated effector memory cells re-expressing CD45RA (TEMRA) cells (**Supplementary Figure 5A**) compared with spike-specific CD4^+^ T cells (**Supplementary Figure 4B**). However, these phenotypes remained largely stable during the memory phase, with older adults exhibiting a tendency toward higher TEMRA proportions in AIM^+^ CD8^+^ T cells (**Supplementary Figure 5A**).

To further characterize CD8^+^ T-cell responses, we examined the expression levels of programmed cell death 1 (PD-1), which is expressed on activated and exhausted T cells (57). Each vaccination induced PD-1 expression on spike-specific AIM^+^ CD8^+^ T cells, and PD-1 expression tended to gradually decrease toward 6 months post following vaccination in both groups (**Figure 4F**). This trend suggests that PD-1 expression may reflect CD8^+^ T-cell activation. Notably, PD-1 expression was lower in older adults than in adults during the primary responses (Post1 and Post2) as well as during the memory response (Post3) (**Figure 4F**, **Supplementary Figure 5B**). Therefore, older adults may exhibit impaired CD8^+^ T-cell activation, which is not ameliorated even after the booster dose.

Taken together, these results strongly suggested that older adults exhibit lower memory CD8^+^ T-cell responses, likely because of impaired activation of spike-specific memory CD8^+^ T cells.

### 3.4 Ameliorated memory B cell response following the third dose is associated with an enhanced antibody response in older adults

**Figure 5:**
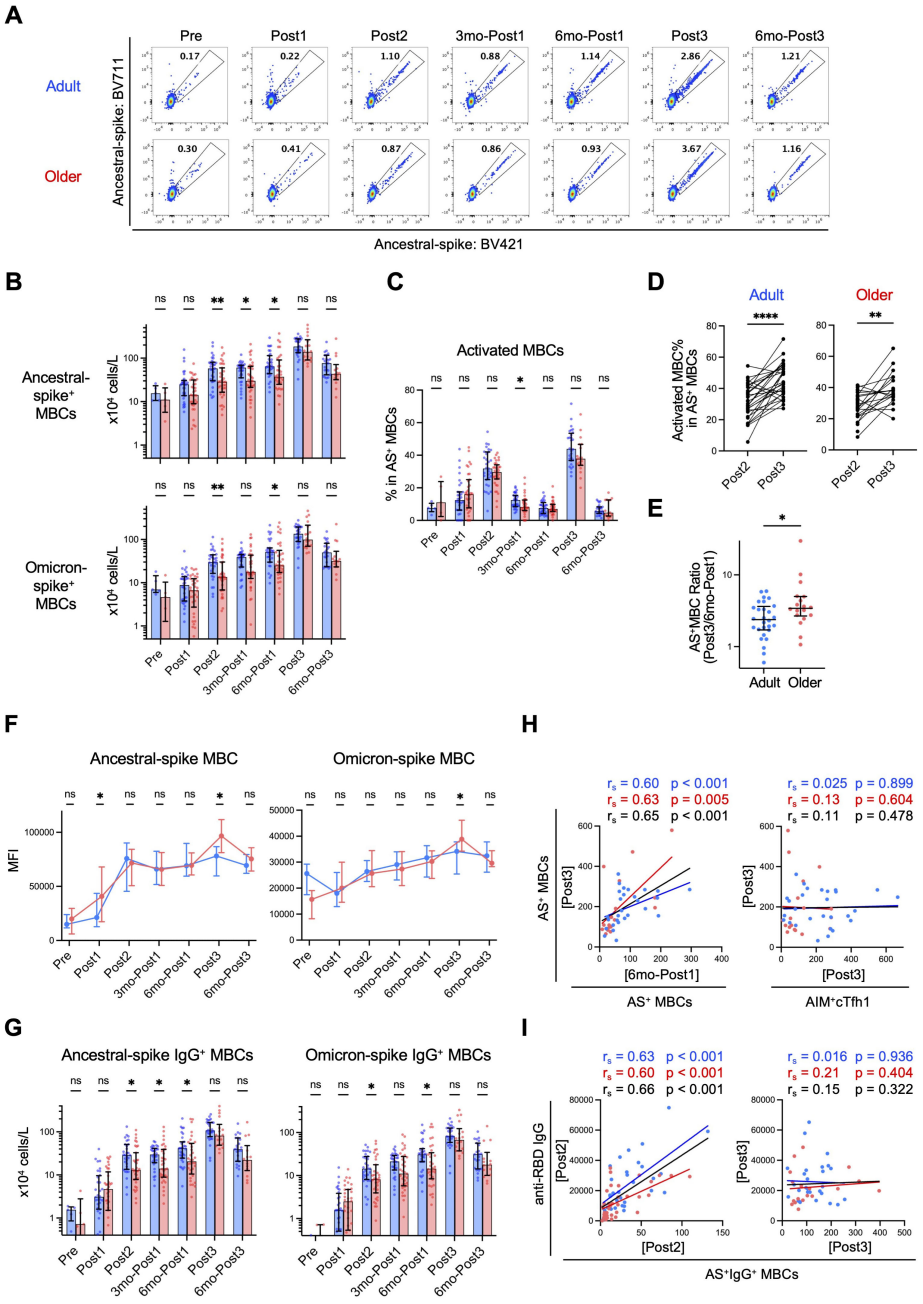
Spike-specific memory B cells (MBCs) are induced to comparable levels in adults and older adults after third-dose vaccination. **(A)** Representative flow-cytometric data of ancestral spike-binding MBCs at each time point. **(B)** Absolute numbers of ancestral spike- or Omicron spike-binding MBCs in blood at each time point. Sample numbers were as follows: 5 (Adults) and 5 (Older adults) (Pre), 32 and 35 (Post1), 31 and 35 (Post2), 31 and 35 (3mo-Post1), 31 and 34 (6mo-Post1), 28 and 18 (Post3), and 23 and 14 (6mo-Post3), respectively. **(C)** Frequency of activated (CD21^–^CD27^+^) cells in Ancestral spike (AS)-binding MBCs. **(D)** Frequency of AS-binding activated MBCs after the second dose (Post2) and third dose (Post3). **p < 0.01, ****p < 0.0001 (Wilcoxon matched-pairs signed rank test). **(E)** Ratio of AS-binding MBCs after the third vaccination (Post3) to that six months after the first dose (6mo-Post1). **(F)** MFIs of AS-BV421 or OS-BV785 among spike-binding MBCs. Dots and error bars indicate medians and IQRs, respectively. **(G)** Absolute numbers of AS- or OS-binding IgG^+^ MBCs in blood at each time point. **(H)** Correlation between the number (x10^4^ cells/L) of AS-binding MBCs after the third dose (Post3) and the number of AS-binding MBCs (x10^4^ cells/L) or AIM^+^cTfh1 cells (x10^3^ cells/L) at the indicated time point. **(I)** Correlation between the number of AS-binding IgG^+^ MBCs (x10^4^ cells/L) and the concentration of anti-RBD IgG (AU/mL) at the indicated time point. **(B, C, G)** Each dot represents the value of each subject. Bar graphs indicate medians, error bars indicate IQRs. **(B, C, E–G)** Blue and red represent adults and older adults, respectively. *p < 0.05, **p < 0.01, ns, not significant (Mann–Whitney test). **(H, I)** Spearman’s rank correlation (r_s_) was used to identify correlations between two variables, and regression lines were drawn based on linear regression analysis. Blue, red, and black characters represent the results of adults, older adults, and both groups, respectively.

As T-cell responses in older adults were lower than those in adults even after the third dose (**Figures 3**, **4**), we investigated the memory B cell responses. The numbers of total and naïve B cells in both groups were largely comparable throughout the study (**Supplementary Figures 6**, **7A**), whereas those of MBCs, including IgM^+^ and IgG^+^ MBCs, were lower in older adults than in adults (**Supplementary Figure 7B**). Spike-specific MBC numbers increased following the first and second doses (Post1 and Post2) in both groups (**Figures 5A, B**, **Supplementary Figure 6**) but were lower in older adults than in adults after the second dose (Post2) (**Figures 5A, B**), which is consistent with a previous finding (11). Thereafter, spike-specific MBCs increased over time in both groups, and the difference was largely maintained over 6 months after the first dose (6mo-Post1) (**Figures 5A, B**). As previously reported (58), the third dose of vaccination caused a further increase in spike-specific MBCs in both groups, and notably, spike-specific MBC numbers in older adults reached levels comparable with those in adults (**Figure 5B**). Similar trends were observed in MBCs expressing BCRs that bind to the spike protein of the Omicron variant (**Figure 5B**). The third vaccination induced a significant increase in activated (CD21^–^CD27^+^) spike-specific MBCs in both groups (**Figure 5C**), and the percentage was higher than that after the second dose in both groups (**Figure 5D**). Moreover, the increase in ancestral spike-specific MBCs from before (6mo-Post1) and after the third dose (Post3) was significantly higher in older adults than in adults (**Figure 5E**). Of note, unlike the second dose, the third dose did not result in a further increase in spike-specific MBCs over the 6 months following vaccination (**Figure 5B**), which implies that the memory B cell responses plateaued after the third dose in both groups. Therefore, spike-specific MBCs in older adults, as in adults, were well activated and proliferated after the third dose, reaching similar levels and plateauing.

Next, we evaluated the affinity maturation of BCRs by examining the kinetics of the mean fluorescence intensity values of fluorescence-labeled antigens as indicators (43–46). The affinity of BCRs to ancestral and Omicron spike proteins gradually increased after the first dose and almost plateaued after the second dose, with no significant differences between adults and older adults, although after the third dose, older adults had higher mean fluorescence intensity values (**Figure 5F**). Next, we investigated class-switch recombination by detecting IgG^+^ spike-specific MBCs. Following the initial vaccination, older adults had lower levels of spike-specific IgG^+^ MBCs than adults as previously reported (11); however, after the third booster dose, the number of spike-specific IgG^+^ MBCs in older adults reached the same level as that in adults (**Figure 5G**). These results strongly suggested that affinity maturation of BCRs occurred at comparable levels in both groups from the primary response to the memory response, and impaired IgG class-switch recombination in older adults was ameliorated following booster vaccination.

Correlation analysis revealed that the number of ancestral-spike-specific MBCs after the third dose (Post3) correlated well with that before the third dose (6mo-Post1), but not with the number of AIM^+^cTfh1 cells after the third dose (Post3) (**Figure 5H**), which suggest that B-cell memory response is less dependent on T-cell help. On the other hand, the anti-RBD IgG titer and ancestral-spike-specific IgG^+^ MBC number correlated after the second dose (Post2), but not after the third dose (Post3) (**Figure 5I**), suggesting that the increase in antigen-specific memory B cells reached a plateau and that some inhibitory mechanisms may be activated following the third dose.

In conclusion, booster vaccination enhances B cell responses in older adults, and thus, the memory B cell response, rather than the memory T cell response, may play a major role in their ameliorated IgG response after the booster shot.

## 4 Discussion

We found that the effects of booster mRNA vaccination on memory responses differed between T cells and B cells in humans. Memory T-cell responses remained lower in older adults even after the booster vaccination. However, impaired B-cell responses in older adults were ameliorated upon booster vaccination, and memory B-cell responses were comparable between the two groups, highlighting the cellular mechanisms underlying enhanced antibody responses in older adults by booster vaccination.

Anti-RBD IgG titers after the second doses (in the primary response) were lower in older adults, whereas those after the third dose (in the memory response) were similar to those in adults, which is consistent with previous findings (13). We found that cTfh1 cells, which facilitate antibody responses, were fewer in older adults than in adults even after the third booster vaccination, and no phenotypic changes, including senescence or exhaustion, were observed. In addition, spike-specific CD8^+^ T-cell responses in older adults were also fewer than those in adults after the third dose. The effects of additional (≥4) vaccinations remain to be examined. These results strongly suggest that enhanced antibody responses in older adults after a third booster dose are not due to ameliorated T-cell responses in the memory phase.

The finding that older adults produced IgG at similar levels as adults following the third dose despite their impaired T-cell memory responses seems to be contradictory. However, we found that B-cell responses in older adults are enhanced by booster vaccination. In the primary response, the frequencies of spike-specific MBCs in older adults were lower but increased to levels similar to those in adults in the memory response following the third dose. Activated MBC frequencies after the third dose were higher than those in the primary response in both groups, suggesting efficient MBC activation and proliferation even in older adults. Moreover, BCR affinities to ancestral or Omicron spike proteins were largely comparable between the two groups throughout the observation period, implying that somatic hypermutation, which broadens the diversity of antibody responses, occurs at equivalent levels in MBCs from adults and older adults. In addition, following the third booster dose, spike-specific IgG^+^ MBC in older adults reached levels similar to those in adults. Although the mechanisms underlying the higher increase rates in antibody titers and MBC numbers in the memory phase in older adults remain unclear, higher pre-existing antibody levels before booster vaccination in adults may be associated with the lower fold increases in recall B-cell responses, probably because of epitope masking and/or inhibitory Fc receptor-mediated apoptosis of MBCs and plasma cells, as suggested previously (58–61). Together, these results indicate that older adults can ameliorate impaired B cell responses but not T cell responses in the memory phase. It remains unclear whether this is due to the nature of the mRNA-induced vaccine response and/or the general immune characteristics of older adults (4). However, considering that T cells are more susceptible to the effects of aging than B cells (30), the difference may be mainly due to intrinsic qualitative and quantitative changes in the T-cell compartment with age. Nonetheless, this does not necessarily imply that the booster vaccination is ineffective in older adults; it may be more accurate to say that memory T cells are at least being maintained at a certain level. To address this, it will be important to compare the number of memory T cells and the protective effects against infection and severe disease between those who received the booster and those who did not.

Previous studies, including ours, have revealed a correlation between the IgG titer and MBC or cTfh cell frequency in the primary response (12, 54, 62). However, we found no correlation between these parameters in the memory responses after the booster dose. Although the need for CD4^+^ T-cells in memory B-cell responses remains controversial (39, 63), studies have demonstrated that human MBCs are activated only in the presence of Toll-like receptor signals (40, 41, 64), suggesting a low dependency on antigen stimulation and T-cell help. Considering that the mRNA-lipid nanoparticle vaccine efficiently stimulates the innate immune system (65), the memory B cell response, which may be well maintained in older adults, likely contributes to the improvement in the antibody response after the third vaccination in older adults. It is also possible that an adequate number of cTfh1 cells exists to induce memory responses even in older adults and that cTfh cells act in a threshold-dependent manner rather than showing a direct correlation. In any case, the effectiveness of antibodies may be affected by viral mutations (66). Therefore, efforts to develop vaccines that improve T-cell responses, in which antigen recognition is less affected by mutations (66), are needed. Importantly, our results indicate that individuals who exhibit poor T-cell responses after initial vaccination tend to also show lower memory responses. Notably, the lower expression of PD-1 on spike-specific CD8^+^ T cells of older adults in the early phase following vaccination provides clear evidence of impaired memory CD8^+^ T cell activation *in vivo.* Therefore, in the future, the enhancement of the CD8^+^ memory T-cell response, which directly eliminates virus-infected cells and is more strongly affected by age, may provide better and long-term protection against mutant viruses in older adults.

This study had several limitations. First, we analyzed antigen-specific memory T-cell and B-cell responses in a limited number of donors (<65 years: n = 32; ≥65 years: n = 35), although the samples used in this study largely represented the tendencies in the primary responses of the original cohort (<65 years: n = 107; ≥65 years: n = 109) (12). Second, individuals ≥65 years of age are commonly defined as older adults, but there is no clear medical or biological evidence to support this definition. Third, some infected individuals may not have been excluded from the analysis due to the sensitivity of anti-nucleocapsid antibody or fluctuations in antibody levels over time (67, 68). Moreover, our results highlighted not only age-related differences but also substantial individual variability, which underscores a larger issue that requires further investigation. Fourth, we did not analyze antigen-presenting cells, which are also critical for vaccine-induced immunity (65). In addition, we only investigated spike-specific T and B cells in peripheral blood; therefore, it remains unclear whether these cell types differ in secondary lymphoid organs or peripheral tissues between adults and older adults. In particular, recent studies have highlighted the differences in phenotypes and functions between circulating and GC Tfh cells (69–73). Finally, we only provided evidence of associations of T-cell and B-cell responses with antibody responses. Further studies are warranted to investigate potential causal relationships between these parameters.

In conclusion, we demonstrated that COVID-19 mRNA booster vaccination significantly improves B-cell responses, but not T-cell responses in older adults, revealing a differential effect of repetitive vaccination on B-cell and T-cell memory responses with age. This study provided insights for the development of more efficient vaccines and establishment of vaccine schedules suitable for older populations.

## Data Availability

The raw data supporting the conclusions of this article will be made available by the authors, without undue reservation.
