# Supplementary material for: Booster COVID-19 mRNA vaccination ameliorates impaired B-cell but not T-cell responses in older adults
Source: Front Immunol. 2024 Dec 9;15:1455334. doi: 10.3389/fimmu.2024.1455334 (PMC11663736; doi:10.3389/fimmu.2024.1455334)
Supplement: Supplementary file 1 [file Table1.docx]

Supplementary Material

# Supplementary Figures

**Supplementary Figure 1. Samples used for antibody response analysis.** The original cohort comprised 107 adults and 109 older adults. The follow-up cohort comprised 103 adults and 105 older adults. Exclusion criteria were applied to the samples used for antibody response analysis, and the number of donors who met all criteria are shown. Samples of donors who met the exclusion criteria were excluded at the time of the respective event or thereafter. Some participants met several exclusion criteria. History of COVID-19 infection was defined by self-report or SARS-CoV-2 anti-nucleocapsid antibody titer ≥0.8.

**Supplementary Figure 2. PBMC samples for the analysis of memory T-cell and B-cell responses**. (A) Thirty-two adults and 35 older adults for whom PBMC samples were available at all time points (Post1, Post2, 3mo-Post1, 6mo-Post1, Post3, and 6mo-Post3) were selected for flow-cytometric analysis. Five samples were randomly selected from adults and older adults for analysis before vaccination (Pre) because of limitations in terms of cell stock availability. Samples of donors who met the exclusion criteria were excluded at the time of the respective event or thereafter. Some participants met several exclusion criteria. (B) Anti-RBD IgG and IgM antibody concentrations in the subjects selected for flow-cytometric analysis. Each dot represents the serum titer of each subject. Center lines and error bars indicate medians and IQRs, respectively. Dashed and dotted lines indicate cutoffs and LODs, respectively. Blue and red represent adults and older adults, respectively. ***p* < 0.01, ****p* < 0.001, ns; not significant (Mann–Whitney test).

**Supplementary Figure 3. Gating strategies for spike-specific T cells.** Gating strategy used to identify spike-specific T cells and T-cell subsets in AIM assays. Lymphocytes were gated based on FSC-A and SSC-A. Doublets were gated out based on FSC-A and FSC-H. Live cells were gated as Ghost Dye Red 710^–^. T cells were gated as CD3^+^ cells and further divided into CD4^+^ and CD8^+^ cells. Differentiation status was defined as naive and stem cell memory (Na/Tscm: CD45RA^+^CCR7^+^), central memory (CM: CD45RA^-^CCR7^+^), effector memory (EM: CD45RA^–^CCR7^–^), or terminally differentiated effector memory cells re-expressing CD45RA (TEMRA: CD45RA^+^CCR7^–^). Circulating follicular helper T (cTfh) cells were gated on CD45RA^–^CXCR5^+^ in CD3^+^CD4^+^ cells. cTfh cells were further divided into 1 and 17 subsets based on CXCR3 and CCR6 expression. Red dots gated on OX40^+^CD137^+^ cells in CD4^+^ cells or CD69^+^CD137^+^ cells in CD8^+^ cells indicate AIM^+^ cells.

**Supplementary Figure 4. Analysis of spike-specific CD4^+^ T cells.** (A) Absolute numbers of AIM^+^ (OX40^+^CD137^+^) CD4^+^ cells in blood. Each dot represents the value of each subject. Bar graphs indicate medians, error bars indicate IQRs. (B) Frequency of the subsets in spike-specific CD4^+^ T cells at each time point. Dots and error bars indicate medians and IQRs, respectively. (C) Absolute numbers of spike-specific CD4^+^ T cells against spike of the ancestral (circle) or Omicron strain (square) in blood after the second (Post2) and third dose (Post3). ns; not significant (Wilcoxon matched-pairs signed rank test). (A, B) Blue and red represent adults and older adults, respectively. **p* < 0.05, ns; not significant (Mann–Whitney test).

**Supplementary Figure 5. Characterization of spike-specific CD8^+^ T cells.** (A) Frequency of the spike-specific CD8^+^ T-cell subset at each time point. Dots and error bars indicate medians and IQRs, respectively. Blue and red represent adults and older adults, respectively. **p* < 0.05, ****p* < 0.001, ns; not significant (Mann–Whitney test). (B) Representative flow-cytometric plot of PD-1 on CD8^+^ T cells. Blue and red histograms indicate PD-1 expression on AIM^+^CD8^+^ T cells of adults and older adults, respectively. Gray-shaded histograms indicate PD-1 expression on total naive CD8^+^ T cells.

**Supplementary Figure 6. Detection of spike-binding MBCs.**  Gating strategy used to identify spike-binding B cells. B cells were gated as Ghost Dye Red 710^–^CD19^+^ cells. Naive B cells were defined as IgD^+^CD27^–^ B cells. Non-naive B cells were further divided into MBCs (CD38^Low/–^CD20^+^) and plasmablasts (PBs: CD38^+^CD20^–^). MBCs were further defined as resting (CD21^+^CD27^+^), activated (CD21^–^CD27^+^), CD27^low^ (CD21^+^CD27^–^), and atypical (CD21^–^CD27^–^). IgM^+^ or IgG^+^ MBCs were defined as IgM^+^IgG^–^ or IgM^–^IgG^+^, respectively. To detect spike-specific MBCs, non-specifically binding B cells (decoy-biotin/SAv-BV510^+^) were gated out. Spike-binding MBCs were identified based on the binding to ancestral spike (decoy-biotin/SAv-BV510^–^ancestral-S/BV421^+^ancestral-S/BV711^+^) or Omicron spike (decoy-biotin/SAv-BV510^–^Omicron-S/BV605^+^Omicron-S/BV785^+^). Spike-binding MBCs were further analyzed for the activation status and immunoglobulin isotype.

**Supplementary Figure 7. Numbers of total naive and memory B cells.** (A) Absolute numbers of total and naive B cells in blood at each time point. (B) Absolute numbers of total, IgM^+^, and IgG^+^ MBCs in blood at each time point. (A, B) Each dot represents the cell number of each subject. Bar graphs indicate medians, error bars indicate IQRs. Blue and red represent adults and older adults, respectively. **p* < 0.05, ***p* < 0.01, ****p* < 0.001, *****p* < 0.0001, ns; not significant (Mann–Whitney test).

# Supplementary Tables

| Antigen | Fluorochrome | Clone | manufacturer | Dilution |
| --- | --- | --- | --- | --- |
| PD-1 | BV421 | EH12.2H7 | BioLegend | 1:100 |
| CD8 | BV570 | RPA-T8 | BioLegend | 1:500 |
| CD69 | BV650 | FN50 | BioLegend | 1:166 |
| CXCR5 | BV750 | J252D4 | BioLegend | 1:50 |
| CCR6 | FITC | G034E3 | BioLegend | 1:100 |
| CD4 | AlexaFluor532 | RPA-T4 | Invitrogen | 1:100 |
| OX40 | PE | Ber-ACT35 | BioLegend | 1:100 |
| CD45RA | PerCP-Cy5.5 | HI100 | BioLegend | 1:100 |
| CD3 | PerCP-eF710 | OKT3 | Invitrogen | 1:250 |
| CD137 | PE-Cy7 | 4B4-1 | BioLegend | 1:100 |
| CXCR3 | APC | G025H7 | BioLegend | 1:50 |
| CCR7 | APC-Cy7 | G043H7 | BioLegend | 1:100 |

AIM assay

| Antigen | Fluorochrome | Clone | manufacturer | Dilution |
| --- | --- | --- | --- | --- |
| IgM | Pacific Blue | MHM-88 | BioLegend | 1:2000 |
| CD19 | BV750 | HIB19 | BioLegend | 1:200 |
| CD20 | FITC | 2H7 | BioLegend | 1:1000 |
| CD38 | PE-Dazzle594 | HIT2 | BioLegend | 1:200 |
| CD27 | PerCP-Cy5.5 | O323 | BioLegend | 1:200 |
| IgD | PE-Cy7 | IA6-2 | BioLegend | 1:2000 |
| CD21 | APC | Bu32 | BioLegend | 1:200 |
| IgG Fc | APC/Fire750 | M1310G05 | BioLegend | 1:200 |

B cells

**Supplementary Table 1.** Antibody list used for flow cytometry
